# Annotations

**Published:** 1887-12-31

**Authors:** 


### ANNOTATIONS.

**Progress in Co-operation.**

THAT co-operation can do much more and better public work than isolation is well shown by the history and present position of The Hospitals Association. The Association has had a "local habitation and a name" since 1883, but it was potentially in existence, and tentatively at work, for two or three years before that time. There is always danger of voluntary associations, which are not immediately in contact with any public work, and which have only such duties to perform as they may themselves assume of their own motion, degenerating into mere "talking clubs"—clubs which give to practical subjects a purely academic consideration, without initiating or carrying out any really useful work. The council and members of The Hospitals Association, in view of the extensive field of practical effort which lay open to them, have always felt that they must do something to justify their existence and to maintain the confidence of the large number of important institutions represented by them. A glance at the present position of the Association and of the operations now in progress, or already completed, in connection with it, will show how successfully these great and important aims are being carried out. At the present time there are directly represented in the membership of the Association no fewer than 140 hospitals and other charitable institutions, a large proportion of these being in London, and many others in Scotland, Ireland, Wales, and the provinces. During the year now closing a great impetus has been given to the Association and its influence by the steady co-operation of this journal with all its movements, so that not only has there been a marked development in its activity and in the public interest taken in its operations, but the number of members has increased in the year by nearly a third, there being 219 on December 31st, as against 156 on January 1st. Among the new members added, or to be added, are—The Earl Sondes, Sir Edmund Currie, Mr. F. C. Carr-Gomm, chairman of the London Hospital; Mr. W. Adams, F.R.C.S., chairman of the Highgate Infirmary; Mr. F. Cleeve, chairman of the Seamen's Hospital; Mr. S. H. Newson, of the North Infirmary, Cork; Mr. F. Browne, of the Isle of Man Hospital; Mr. R. P. Barrow, of the Drapers' Company; Mr. George Weston, of the Fishmongers' Company; Mr. Percy Wigram, Treasurer of the East London Nursing Association; Mr. John Croft, F.R.C.S., of St. Thomas's Hospital; Mr. George King, Actuary of the Atlas Insurance Company; with representatives of hospitals in Glasgow, Norwich, and many other large towns. Of the work which is now in hand the public are already familiar with the large and comprehensive scheme of sick insurance and the means of retirement for aged and disabled nurses which everybody knows as the *National Pension Fund*. Another scheme, equally comprehensive, and one capable of rendering incalculable service to the sick poor and those who feel it incumbent to provide hospital accommodation for them, is that standing in the name of Sir Andrew Clark, and called "The Sick and the Hospitals." A "Nurses' Registration" scheme, the value of which will be enormous both to those who employ nurses and to the nurses themselves, is in a forward state of organisation, and has been practically opened to the nursing world. This scheme it is hoped may be carried out under the auspices of the General Medical Council, or some representative body, thus securing to nurses a recognised public position which ten years ago the most sanguine would never have ventured to predict. "Diet Tables for Hospitals," "Information on Nursing and Domestic Management," "Rules and Methods for the Training of Nurses," and other kindred topics, are all being dealt with as time and opportunity allow, and give assurance of an intelligent and far-reaching programme of the highest possible public and personal value. In view of such a record of progress and serious purpose, it is earnestly to be hoped that the hospitals and their responsible officials, as well as the medical and nursing professions and the charitable public, will join unanimously and warmly with the Association in its strenuous and persevering efforts for the general good.

**St. John's Hospital for Skin Diseases.**

THE affairs of St. John's Hospital for Diseases of the Skin have often engaged the attention of the public, and during the last few weeks several letters, circulars, and notices have reached us. The managers appear to attach much importance to their circular of the 19th instant, marked "official," in which they declare that the special board of governors summoned for the 20th inst. was issued without the authority of any person competent to convene such a meeting. The meeting was called in virtue of a requisition to the treasurer signed by nine governors. If the independent governors cannot move the treasurer, or whoever the proper official may be, to call a special meeting within a reasonable time, the bye laws and regulations must be unusually defective. We should not have alluded to the subject until after the meeting of the governors fixed for January 18th next, had we not received a printed letter of an unusual character from the hon. solicitors to the hospital, which seems to have been addressed to the Press generally, and which, if it has any meaning at all, contains a distinct threat in the case of our publishing a report "supplied to us for publication either by our own reporter or by a private individual." Any editor with a sense of self-respect and a due regard for the public interest, is bound on public grounds to urge every governor of the institution to make a point of attending the meeting on the 18th January next. We feel it our duty to state further that there is but one remedy for the present unpleasant business in connexion with this hospital, and that lies in a full and impartial investigation by a competent committee appointed by the governors. The committee of management, if they attempt to meet the allegations of their opponents, must yield this point, and if they had been wise they would have volunteered it already. Had they shown any sign of being ready to take this step, it would no doubt have been better to have waited for their proposals, but under all the circumstances, no impartial person can wonder if the governors and subscribers show themselves resolved in the last extremity to force the hands of the committee of management. We hope that wiser counsels will prevail, however, and that a properly constituted committee of inquiry may be unanimously appointed at the meeting on the 18th prox. It is not desirable to express any opinion on the matters in dispute without having access to the books and papers, and a full knowledge of all the circumstances from first to last. This fact renders it impossible for any meeting of governors to deal with a case of this kind, except by referring it to a small number of representative and qualified persons, in whom both the public and the governors may place the utmost confidence.

**Children's Dress.**

"WE are all born royal," it has been said, and probably no kingdom boasts such loyal subjects and so few rebels as that of childhood. As it is only courteous and right that we should interest ourselves in all that concerns our rulers, we need express no surprise that one of our most widely-read daily papers should devote a whole column to the discussion of the important question of children's dress. There are of course several ways in which this question can be regarded. There is the point of view of fashion, and that of common sense. It is a pity that the first of these considerations should ever exist apart from the second; but unfortunately, especially for girl children, it does, and as a rule it is in the earliest infancy of these that the compression which is to make them "good figures" begins, to be followed as soon as they can stand on their tiny feet with preparations for making these "neat"—and deformed. It is as a rule useless to preach dress reform to adults. A woman whose foot has been trimmed by twenty years or more of compression to the conventional shape, suffers more from hygienic boots than from the most narrow-toed productions of the average shoemaker; and the result of her attempts to regain the normal anatomical waist are hideous to behold; nature, distorted from her rightful curves and outlines, cannot return to them. But all women who have taken to heart the lessons of physiology, may help to save children from the mistakes that follow a blind adherence to fashion. At present, besides the serious blunders already spoken of in connexion with the dress of little girls, there is a fashion of cutting away the thicker woollen stuff of which the dress is made in front, in order to "let in" a puff of thin silk or lace; that is, in a country where diseases of the respiratory organs are painfully common, it is the custom to make the clothing thinner rather than thicker over the chest, thus inducing colds which may ultimately develop into bronchitis or phthisis. Children's dress is a matter with which fashion should have little to do. Health and comfort should at any rate be the primary if not the sole considerations.

